# Effects of Infill Pattern and Infill Density on the Flexural Properties of Markforged Onyx

**DOI:** 10.3390/polym18172060

**Published:** 2026-08-25

**Authors:** Jonathon Tran, Rachel Shubella, Alexander Hunt

**Affiliations:** Department of Mechanical and Materials Engineering, Portland State University, Portland, OR 97201, USA; tranjon@pdx.edu (J.T.); shubella@pdx.edu (R.S.)

**Keywords:** additive manufacturing, composites, Nylon, Markforged, Onyx, infill pattern, infill density, flexural testing, optical microscopy, scanning electron microscope, ANOVA

## Abstract

Fused Filament Fabrication (FFF) enables the production of lightweight polymer composite components with complex geometries; however, the effects of infill geometry on printing accuracy and mechanical behavior of unreinforced Markforged Onyx remains insufficiently understood. This parametric study investigates the influence of four infill patterns (gyroid, hexagonal, rectangular, and triangular) and three infill densities (30%, 40%, and 50%) on the post-print mass, dimensional accuracy, microstructure and flexural properties of Onyx specimens fabricated using a Markforged Mark Two printer. Experimental measurements were compared with Eiger slicing software predictions, and geometry- and mass-based area moment of inertia correction methods were evaluated. The results show that Eiger consistently overestimated part mass by approximately 8–10% with the largest deviations occurring for the hexagonal infill pattern. Increasing infill density increased flexural strength and stiffness for every infill pattern. The triangular pattern exhibited the highest mechanical performance, whereas the hexagonal pattern produced the lowest due to its larger internal voids and reduced effective material. The mass-based correction method produced flexural properties comparable to the geometry-based approach, providing an alternative method for predicting the mechanical performance of FFF-manufactured Onyx components.

## 1. Introduction

**Figure 1:**
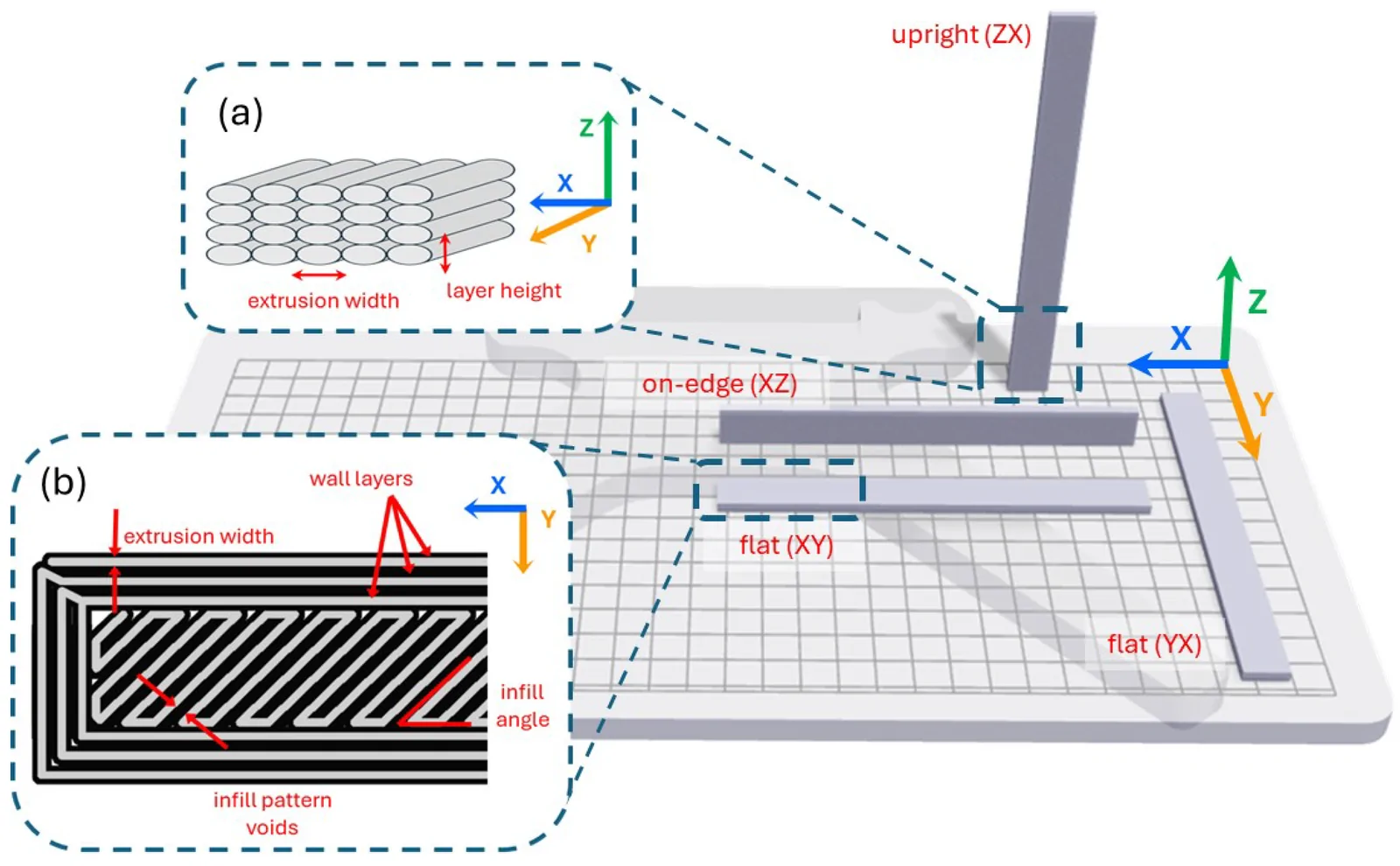
Printer bed with build orientations, build directions and process parameters. (**a**) Process parameters in the ZX-direction. (**b**) Process parameters in the XY-direction.

Fused filament fabrication (FFF) is one of the most widely adopted additive manufacturing technologies because it enables the production of complex geometries with minimal material waste and reduced tooling requirements [1]. Despite its advantages over conventional manufacturing processes, FFF-made parts often have weaker mechanical properties limited by the layer-by-layer deposition process [2,3]. The materials used for 3D printing are mainly thermoplastic polymers, and the FFF process introduces voids between extrusion layers due to the elliptical shape of the extrusion beads that do not fully fill the corners between adjacent extrusions (Figure 1), resulting in anisotropic material properties [4,5,6]. Recent advances have seen the integration of fiber reinforcements, such as carbon fiber, glass fibers, or Kevlar that significantly improve mechanical strength and stiffness while maintaining a lightweight profile [7,8,9,10,11,12,13,14,15,16,17].

This work specifically investigates Markforged Onyx, one of the most popular carbon fiber–nylon composites commercially available at this time [18]. Onyx consists of a nylon matrix reinforced with short, chopped carbon fibers. Onyx can be printed as a filament composite or further reinforced with a continuous fiber to provide additional resistance. It is a high-strength thermoplastic material with excellent surface finish and high resistance to chemical agents compared to most other FFF materials. Significant mechanical properties of Onyx, as reported by Markforged, are summarized in Table 1.

Most prior work on Markforged Onyx prints emphasizes the addition of continuous fiber reinforcement to achieve improved mechanical performance [5,9,19,20,21,22,23,24,25], with only a handful of studies investigating the unreinforced composite material. These studies show that increasing infill density has been shown to increase both volume and tensile strength for rectangular, hexagonal, and triangular infill patterns [26,27,28]. Among these infill patterns, rectangular infill has consistently demonstrated greater increases in mass, volume, and tensile strength compared to hexagonal and triangular infill patterns. However, it has not been reported as to how well parts printed with Onyx match the desired part geometries, percent infill, and expected part mass. It is not clear if the increase in strength is attributable to the increase in volume and mass, or due to the infill geometry directly. Additionally, none of the studies have investigated the gyroid infill pattern that is also available in Eiger at this time. It is also worth noting studies investigating the flexural strength of Onyx parts reported lower flexural strength and modulus values than the Markforged data sheet, but there is no clear consensus on flexural strength and modulus values across these studies [28,29,30,31]. More studies are needed to help determine material strength of the 3D printed parts.

Additionally, it is still not clear how best to calculate part strength for 3D printed parts. The American Society for Testing and Materials (ASTM) test for non-reinforced rigid polymers, ASTM D790, is used for flexural testing [32]. Despite having guidance for tool configuration, geometries and dimensions of test samples, and testing conditions, ASTM D790 does not establish any criterion (of order or quantity) of the production of parts or methods of stress analysis for test samples with hollow interiors. A preliminary study on polylactic acid (PLA) found that considering the real hollow moment of inertia of extruded parts [33] resulted in higher material strength values; however, there is still a lot of variability in their calculations. More studies and additional theory are needed to better predict the strength of parts with partial hollow interiors created by different infill patterns.

This work presents a novel investigation of the effects of the infill pattern and infill density for the unreinforced Markforged Onyx nylon composite by using corrective methodologies for calculating the strength and modulus of hollow specimens. Infill densities of 30, 40 and 50% for each infill pattern are compared. We analyze geometrical properties such as final print mass, infill mass excluding the outer shell, width and depth measurements, and estimated hollow cross-sections. We also measure flexural stiffness and strength using three-point bending tests.

## 2. Materials and Methods

The dimensions of the ASTM D790 flexural test specimens in the XY-direction are 127 mm for length (*l*), 12.7 mm for outer width/base (*B*) and 3.2 mm for outer height/depth (*D*). The test samples are printed using a Mark Two additive manufacturing printer by Markforged. The X, Y, Z labels used for the orientation of the sample (Figure 1) are in accordance with the International Organization for Standardization (ISO) 17295 Standards for Coordinate Systems and Test Methodologies [34]. As stated in the standard, Z denotes the vertical build direction. The X axis is perpendicular to the front of the machine and perpendicular to the Z axis. The Y axis is normal to both the Z and the X axes, with a positive direction defined by using right-hand set coordinates. Five of each sample are manufactured and tested in the XY-direction (flat).

The flexural test specimens are tested for bending strength using a Tinius Olsen 5ST electromechanical testing machine (Tinius Olsen Testing Machine Company, Horsham, Pennsylvania, USA). The samples are placed on the flat edge of the apparatus to perform the 3-point bend test and the deformation is controlled at a loading nose speed of 2 mm/min. The length of the span between the two supports is 51.2 mm.

A dry box is used to store the Onyx filament and ensure that water does not contaminate the nylon base. The nylon is kept within the dry box and fed directly from the unit to the printer. The sample is prepared on an enclosed bed with a garolite surface covered with a thin layer of glue (applied by a glue stick) to promote adhesion to the bed.

The slicing software, Eiger, is closed source and does not allow the user to adjust some printing parameters such as temperature, nozzle movement, infill angle or extrusion speed. The experimental printing parameters utilized in this study can be seen in Table 2.

This work tests the strength of parts manufactured at various infill percentages and with different infill patterns. Eiger has infill density parameters that limit the infill density minimums and maximums for each infill pattern:

- Gyroid infill density can be printed between 28–52%;
- Hexagonal infill density can be printed between 18–62%;
- Rectangular infill density can be printed between 0–92%;
- Triangular infill density can be printed between 28–55%.

**Figure 2:**
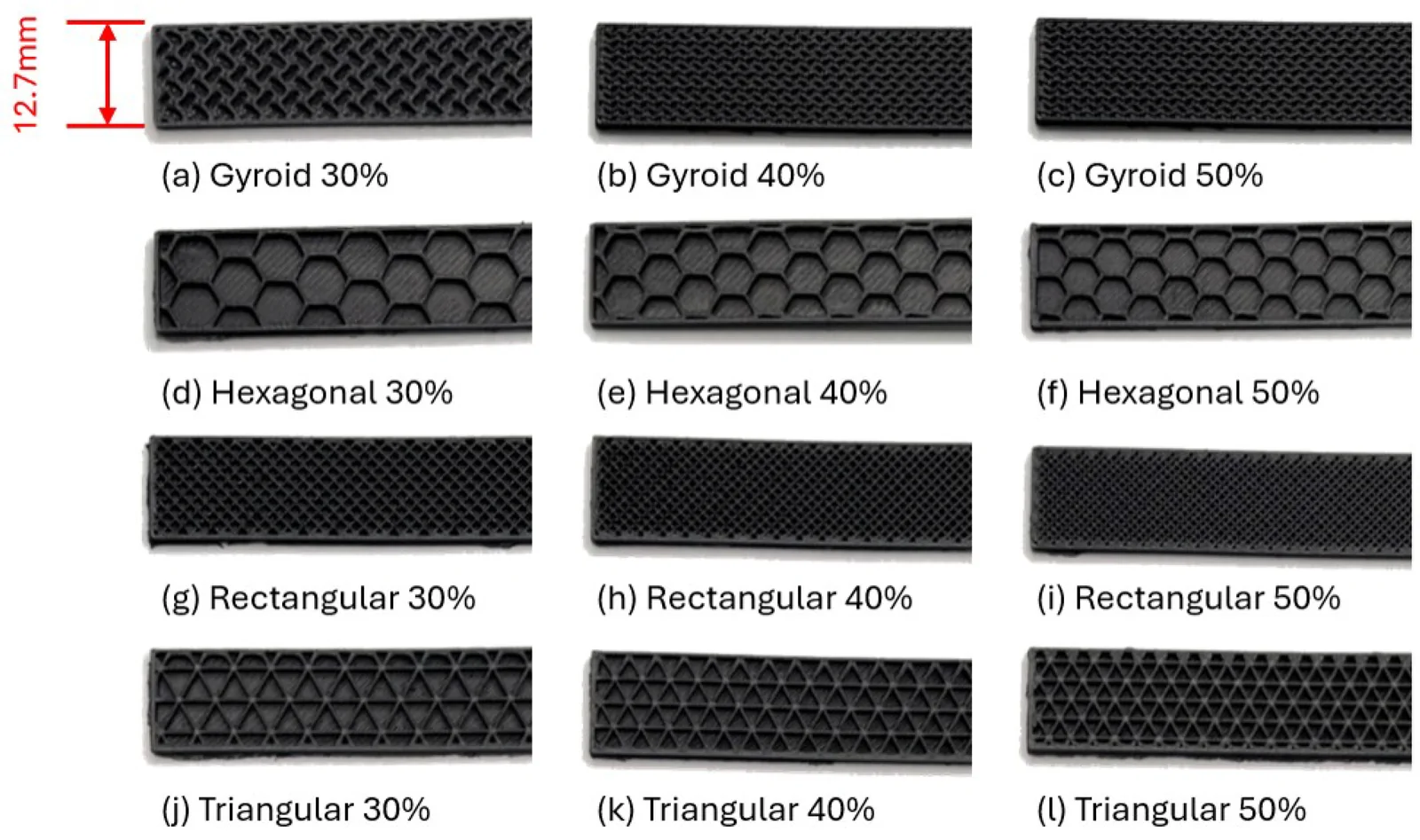
Onyx specimens showing the infill patterns and infill densities investigated (images were taken of specimens which were paused mid-print to allow infill pattern and infill density to be observed).

For comparative consistency, parts are printed in each infill pattern at 30, 40, and 50% infill densities (Figure 2). All samples are fabricated with identical external/outer geometries, consisting of a single outer wall all the way around, a single roof layer, and a single floor layer. Therefore, variations in mechanical performance are mainly attributed to differences in the internal infill structure.

### 2.1. Predicted and Experimental Dimensions, Mass, and Infill Percentage

One aim of this work is to determine how well Eiger predicts the mass, dimensions, and infill percentage of the final part. The desired print dimensions act as the predicted dimensions for the part. Mass prediction is an output of the Eiger software (3.20.160). Eiger also allows users to select infill percentage, and this is used as the predicted infill percentage.

For experimental values, the dimensions of each sample are measured using a Mitutoyo 500-197-30 digital caliper (Mitutoyo Corporation, Kanagawa, Japan) to obtain the outer width (*B*) and outer depth (*D*) dimensions. The mass is obtained using an OHAUS Discovery Semi-Micro and Analytical balance (OHAUS Corporation, Parsippany, NJ, USA).

The experimental infill percentage is found using a mass-based approach. The infill percentage can be calculated as:

(1) $$Infill\%=\frac{m_{infill}}{V_{infill}*\rho }$$

where

(2) $$m_{infill}=m_{part}-m_{shell}$$

where $m_{shell}$ was found by printing and weighing the averages of 5 parts with no infill material.

(3) $$V_{infill}=V_{part}-V_{shell}$$

where the volume of the outer shell consists of the perimeter walls, roof, and floor:

(4) $$V_{shell}=V_{roof}+V_{floor}+2V_{XYwalls}+2V_{YXwalls}$$

and ρ is the density of the material given from the materials data sheet.

### 2.2. Area Moment of Inertia Calculations

This work also investigates how part strength may be better predicted given different infill patterns and infill density values without requiring Finite Element Analysis. The first method for testing uses an approximate moment of inertia in the rectangular hollow beam ($I_{apparent}$), calculated using an effective cross-sectional geometry.

**Figure 3:**
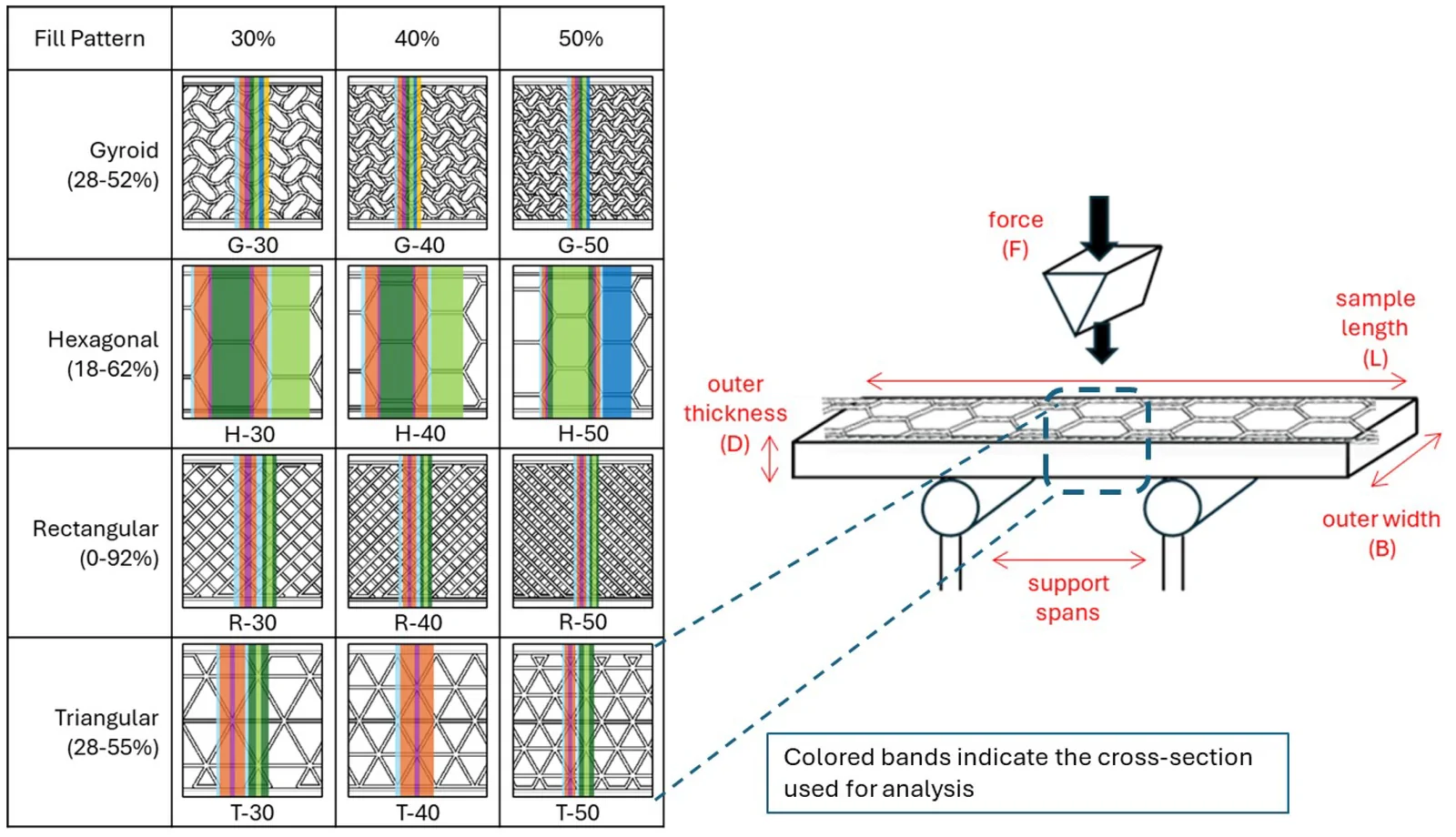
Infill patterns and densities used with cross-sectional areas measured to obtain interior dimensions for 3-point bending test. The colored vertical bands represent the cross-sectional regions with equivalently sized hollow space.

**Figure 4:**
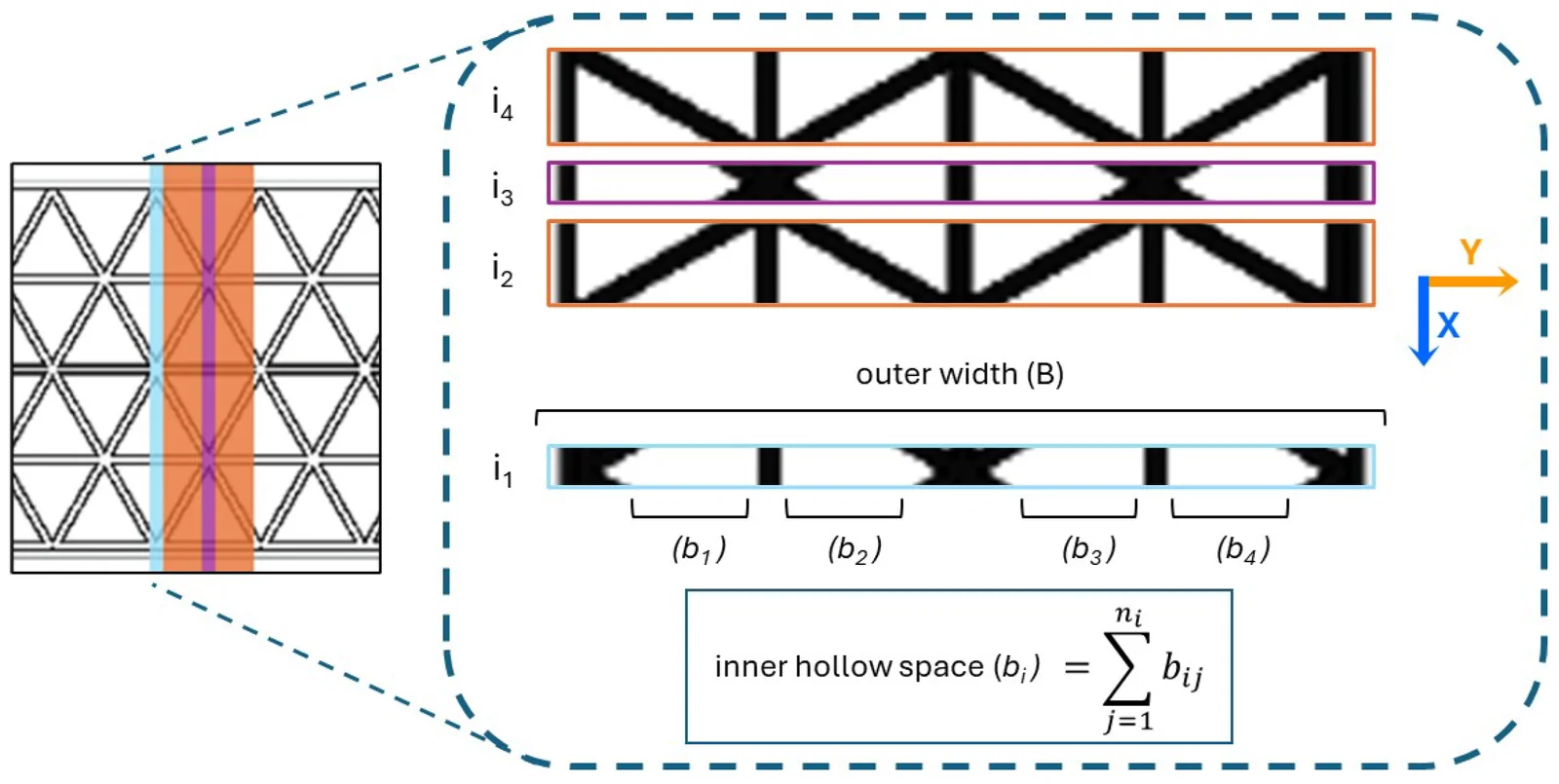
Example image of internal hollow space measurements for triangular pattern at 40%. infill density.

To account for the geometric complexity, a geometry-based method was developed as an analytical model. Each infill pattern is reduced to an equivalent homogenized cross-section represented by a hollow rectangle. The internal structure is characterized by an averaged inner hollow width space ($\bar{b}$) and an inner hollow depth space (*d*). To calculate this equivalent cross-section, the smallest repeating pattern along the length of the part is found first. This pattern is then divided into regions with equivalently sized hollow space (Figure 3, colored sections). Each cross-section may contain a different number of measured regions and internal segments. The hollow width in each of these cross-sections ($b_{i}$) is found by summing the gaps ($b_{ij}$, Figure 4). The regions are then combined through a weighted sum to obtain the average amount of hollow width space, ($\bar{b}$), such that:

(5) $$\bar{b}=\sum_{i=1}^{N}\left[\left(\sum_{j=1}^{n_{i}}b_{ij}\right)w_{i}\right]$$

where *N* is the total number of cross-sections considered, $n_{i}$ is the number of inner hollow width spaces measured in the cross-section *i*, $b_{ij}$ is the width of the *j*th gap in the cross-section *i*, and $w_{i}$ is the percent of space occupied by the cross-section *i*.

The inner depth space is calculated by subtracting the roof and floor layers from the outer depth:

(6) d=D−0.125mm(roof)−0.125mm(floor)

The area moment of inertia for the rectangular solid beam cross sections ($I_{apparent}$) and the rectangular hollow beam cross sections based on geometry ($I_{geo}$) are calculated as follows:

(7) $$I_{apparent}=\frac{BD^{3}}{12}$$

(8) $$I_{geo}=\frac{BD^{3}-\bar{b}d^{3}}{12}$$

This provides a geometry-based framework to compare infill designs without requiring finite element analysis.

### 2.3. Area Moment of Inertia Based on Mass Ratio

The second method used for estimating part strength and stiffness uses a mass ratio. This method uses the ratio between the percent of mass in the printed part with a theoretical 100% infill to adjust the moment of inertia accordingly. The theoretical mass of a completely solid sample was calculated using the following equation:

(9) $$m_{part}=\rho \cdot V_{part}$$

where ρ is the density given by the material data sheet. The adjusted area moment of inertia based on a mass ratio ($I_{m\phi }$) was calculated using:

(10) $$I_{m\phi }=I_{apparent}\cdot \frac{m_{exp}}{m_{part}}$$

### 2.4. Flexural Strength and Strain Calculations

The calculations of mechanical properties using 3-point bend test methods are based on the beam theory for a specimen loaded under uniaxial bending stress. For a simply supported beam under 3-point bending, the flexural strength ($\sigma_{F}$) is calculated from the following equation [35]:

(11) $$\sigma_{F}=\frac{Mc}{I}$$

where *M* is the bending moment, *c* is the distance from the neutral axis, and *I* is the area moment of inertia. The equation for the bending moment is given by the following equation:

(12) $$M=\frac{FL}{4}$$

where *F* is the bending force and *L* is the length between the support spans (51.2 mm). The distance from the neutral axis for a simply supported beam under 3-point bending is given by:

(13) $$c=\frac{D}{2}$$

If we put Equations (12) and (13) into Equation (11), we get the following:

(14) $$\sigma_{F}=\frac{FLD}{8I}$$

The measured flexural strain data was normalized to account for the initial compliance effects associated with sample seating, fixture deformation, and system-level displacement offset. The flexural strain value ($\varepsilon_{f}$) on the results of the bending test is calculated according to the following equation:

(15) $$\varepsilon_{f}=\frac{6\delta D}{L^{2}}$$

The maximum deflection (δ) is calculated using the following equation:

(16) $$\delta =\frac{FL^{3}}{48EI}$$

where *E* is the flexural modulus and *I* is area moment of inertia. The flexural modulus for a solid rectangular beam ($E_{apparent}$), a hollow rectangular beam ($E_{geo}$) and a beam with an adjusted area moment of inertia based on mass ratio ($E_{m\phi }$) for a 3-point bending test can be found by combining Equations (7), (8), and (10) with (16) to get:

(17) $$E_{apparent}=\frac{L^{3}}{4BD^{3}}\frac{F}{\delta }$$

(18) $$E_{geo}=\frac{L^{3}}{4(BD^{3}-bd^{3})}\frac{F}{\delta }$$

(19) $$E_{m\phi }=\frac{L^{3}}{4BD^{3}\left(\frac{m_{exp}}{m_{part}}\right)}\frac{F}{\delta }$$

where $\frac{F}{\delta }$ is the initial slope of the load–deflection curve, calculated using the following equation from the experimental data.

### 2.5. Microstructural Analysis

Microstructural imaging and characterization of the flexural samples for printing quality and surface morphology were performed. Fracture analysis was not performed as none of the samples fractured during 3-point bend testing. For optical microscopy (OM), longitudinal sample cross sections were cut by Dremel, seeded in epoxy resin (Ted Pella, Inc., Redding, CA, USA), ground and polished with 180, 400, 800 and 1200 grit sandpapers sequentially, followed by a 3 μm polycrystalline diamond slurry as described in Table 3. After grinding and polishing the samples, they were placed in a Branson 2510 ultrasonic cleaner (Branson Ultrasonics Corporation, Brookfield, CT, USA) for 15 min to dissipate any lingering air bubbles within the sample’s voids. Scanning electron microscopy (SEM) samples did not require any sputter coating as they are already conductive. SEM sample cross sections were cut longitudinally and transversely by Dremmel and taped onto an SEM mount using carbon tape. Microstructural imaging was performed using an AmScope MU1803 camera (United Scope LLC (AmScope), Irvine, CA, USA) attached to a Nikon Eclipse L200 optical microscope (Nikon Corporation, Tokyo, Japan) and a Hitachi TM4000II scanning electron microscope (Hitachi High-Tech Corporation, Tokyo, Japan).

### 2.6. ANOVA Analysis

An analysis of variance (ANOVA) was performed to develop prediction models and determine the distinguishing factors that influence the experimental bending stresses and moduli. To determine the importance (*F*-value) and significance (*p*-value) of each parameter, values given in the ANOVA table are utilized where DF represents the degree of freedom, SS is the sum of squares, and MS is the mean of squares. If the *p*-value is less than 0.05, the significance of the related term is established as significant.

## 3. Results

The experimental masses of all printed samples were consistently lower than the values predicted by the Eiger slicing software. For gyroid, rectangular, and triangular infill patterns, the total mass of the measured part was approximately 8–9% lower than predicted for all infill densities (Table 4). The hexagonal pattern exhibited a larger variation relative to the predicted values, approximately 6–12%. The internal infill mass ratios for the rectangular pattern had the best accuracy for the true infill percentage, consistently within 2% of the predicted infill percent. Gyroid and triangular patterns were within 4%. The true infill ratio for the hexagonal pattern was significantly off, with a calculation of 16% for the 30% infill, 22% for the 40% infill, and 21% for the 50% infill. These deviations indicate that Eiger consistently overestimates material deposition, and that methodologies used for calculating infill percent for the hexagonal structure are significantly incorrect.

Dimensional measurements also revealed that the printed samples exhibited slight reductions in width and depth compared to the values predicted by Eiger (Table 5). The average of all samples exhibited a decrease in the width and depth measurements by ~0.79% and ~2.88%, respectively. However, the average of all samples exhibited an increase in length measurement of approximately 0.10%.

The calculated area moment of inertia for the hollow cross-sections ($I_{geo}$) increased with increasing infill density for all infill patterns. When accounting for internal void geometry, using Equation (5), the gyroid, rectangular, and triangular patterns exhibited comparable inertia values, while the hexagonal pattern consistently showed the lowest values. The reduced inertia observed in the hexagonal pattern can be attributed to its larger internal void spacing and the actual lower infill density. When comparing $I_{geo}$ to $I_{apparent}$, all infill patterns exhibited a reduced area moment of inertia, relative to the experimental infill density selected.

Using Equation (10), the relative area moment of inertia was calculated as a mass ratio ($I_{m\phi }$). When comparing $I_{m\phi }$ to $I_{apparent}$, all infill patterns also exhibited a reduced area moment of inertia, resulting in values similar to $I_{geo}$.

**Figure 5:**
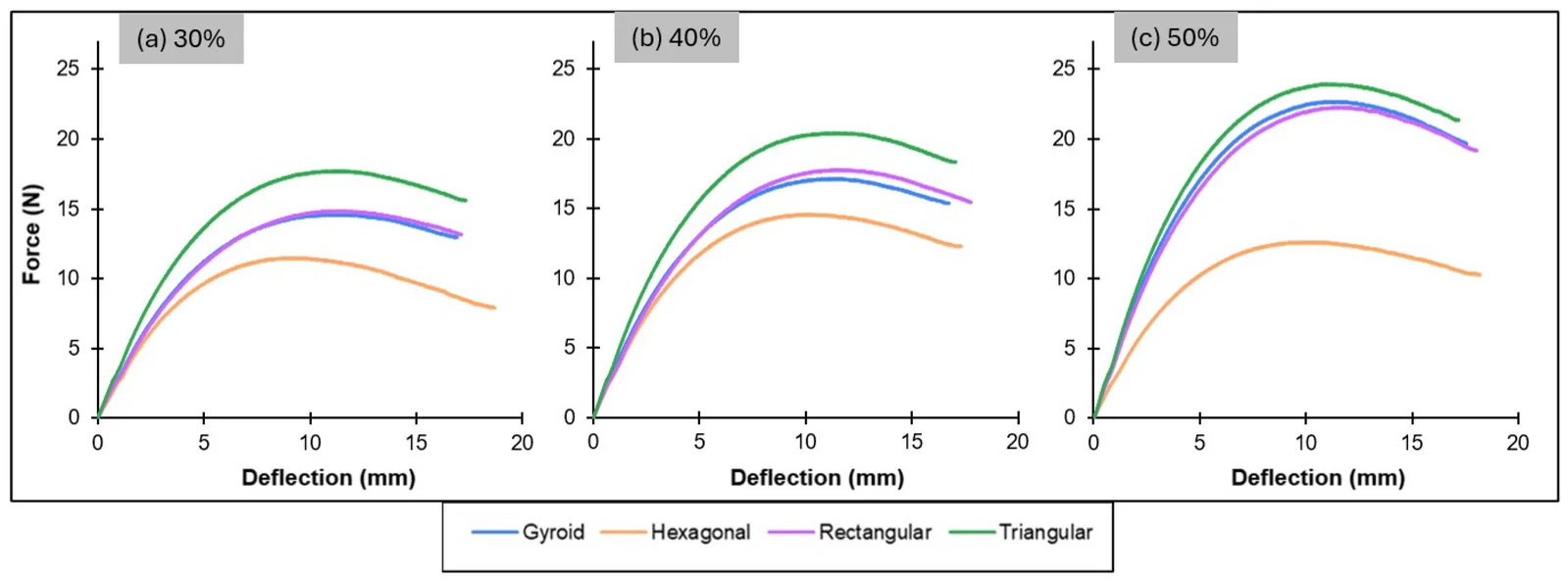
Comparison of the force–deflection curves of the flexural specimens based on an apparent solid cross section at (**a**) 30% infill, (**b**) 40% infill, and (**c**) 50% infill.

The results of the force–deflection curves based on the apparent solid cross section ($\sigma_{apparent}$) indicate that the higher the percentage of infill density for any selected infill pattern, the greater the stiffness and resistance to the load, except the hexagonal parts, which demonstrated reduced strength at 50%. This is due to the fact that the actual hexagonal infill percent was actually lower for the 50% specimens than the 40% specimens. (Figure 5). The triangular pattern exhibits the strongest resistance to the deformation force at all the infill densities studied, followed by the gyroid and rectangular patterns, which exhibit similar flexural strengths. The hexagonal pattern exhibits the lowest resistance to deformation at all infill densities.

**Figure 6:**
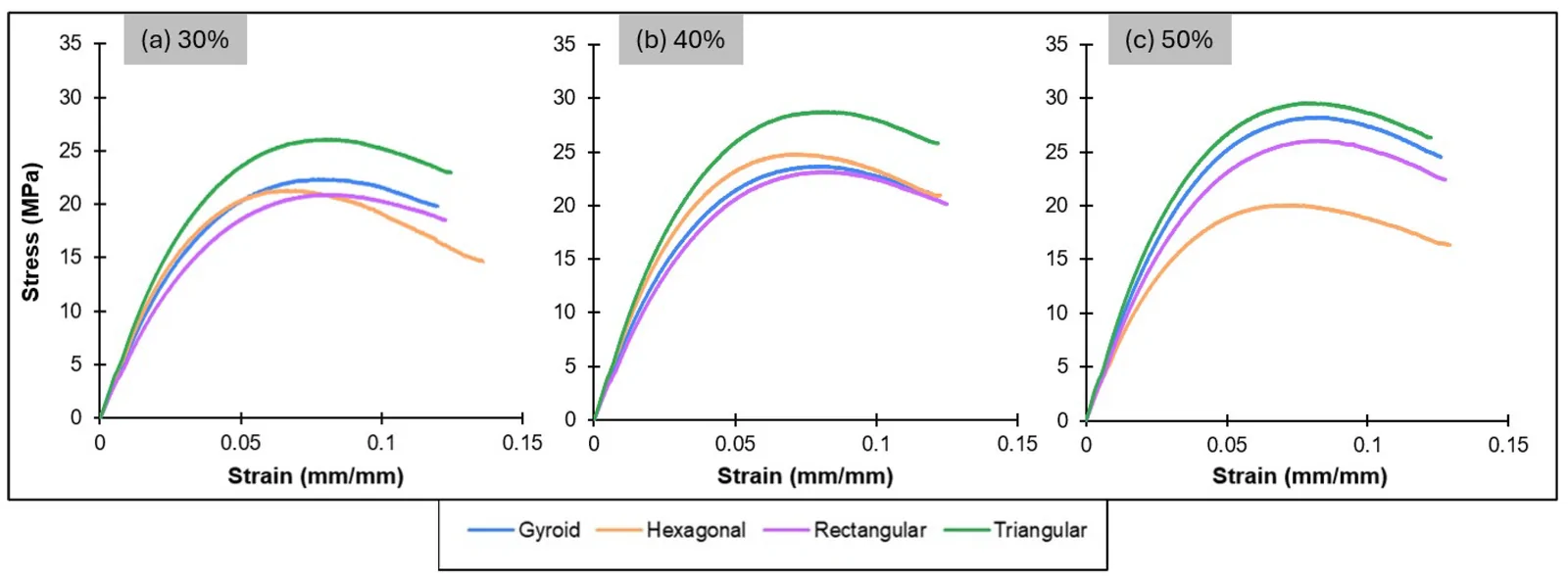
Comparison of the stress–strain curves of the flexural specimens based on a hollow geometry cross section at (**a**) 30% infill, (**b**) 40% infill, and (**c**) 50% infill.

The results of the stress–strain curves corrected for the hollow geometry cross sections show reduced variations (Figure 6). The triangular patterns still demonstrate the strongest resistance at all infill densities tested, with gyroid and rectangular patterns at a little less. However, the hexagonal curves closely match the gyroid and rectangular strengths at 30% and 40%, while showing reduced strength at 50%. The average flexural strength for the geometry specimen population was 24.77 MPa, with a coefficient of variation of approximately 18%.

**Figure 7:**
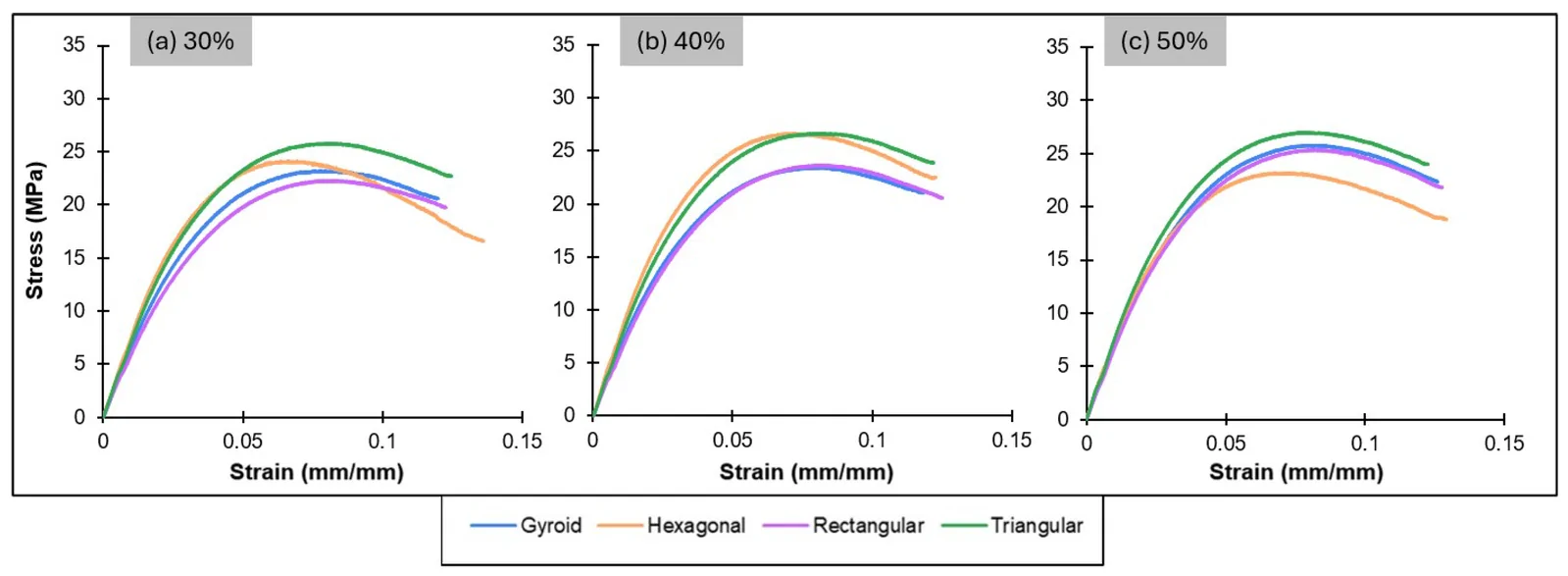
Comparison of the stress–strain curves of the flexural specimens based on a hollow mass ratio cross section at (**a**) 30% infill, (**b**) 40% infill, and (**c**) 50% infill.

**Figure 8:**
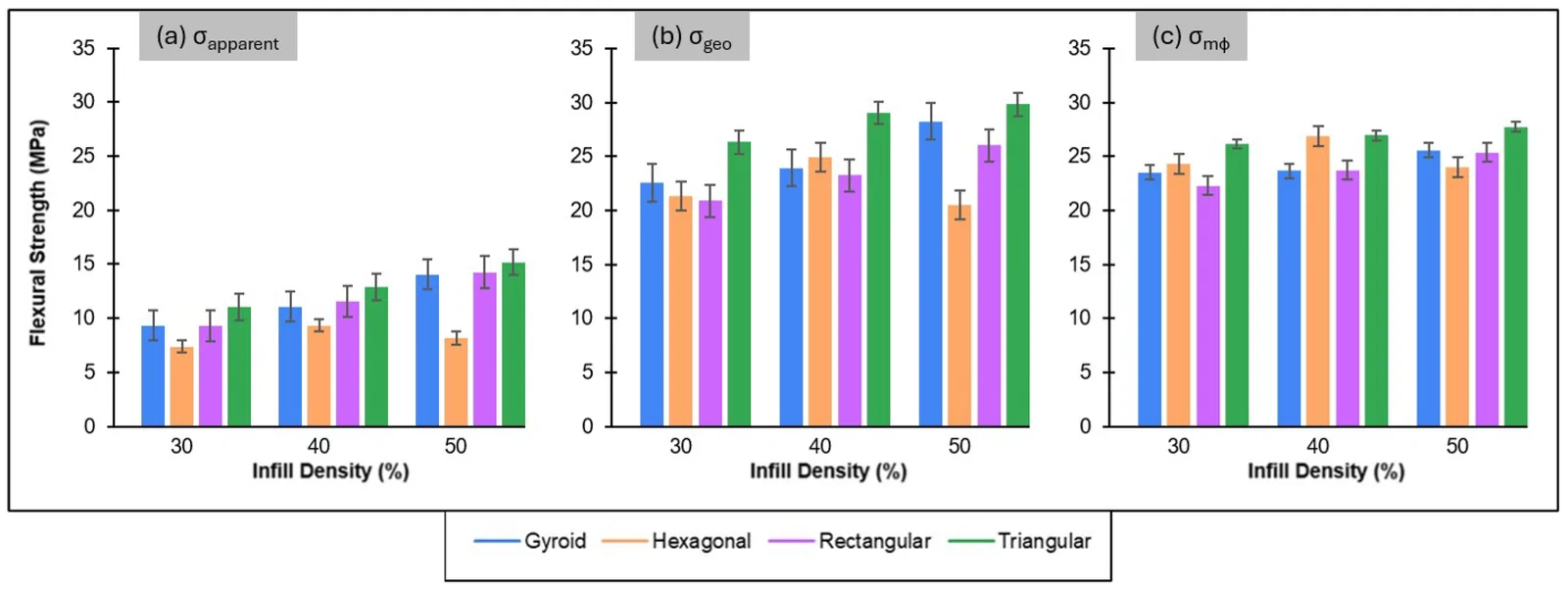
Comparison of the material flexural strength based on a (**a**) apparent solid cross section, (**b**) hollow geometry cross section, and (**c**) hollow mass ratio cross section.

The results of the stress–strain curves corrected for the hollow mass ratio cross section show even less variation between all the specimens (Figure 7). The relative strength of the hexagonal specimens have increased even more, matching triangular at 40% and close to the overall mean at 30% and 50% as well. The average flexural strength for the mass ratio sample population was 24.97 MPa, similar to the geometry samples; however, there is a smaller coefficient of variation in the mass ratio specimens of approximately 14%. A comparison of the material strength as calculated by all three methods can be found in Figure 8.

**Figure 9:**
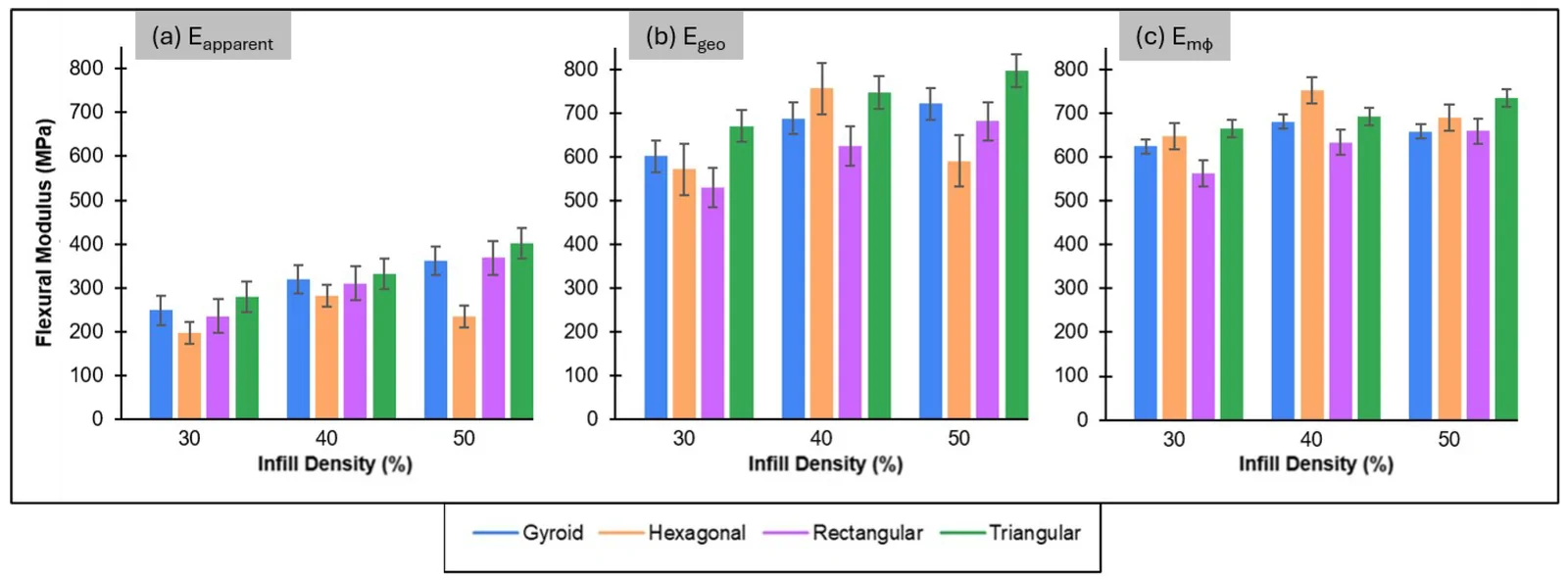
Comparison of the material flexural modulus based on (**a**) apparent solid cross section, (**b**) hollow geometry cross section, and (**c**) hollow mass ratio cross section.

The apparent flexural modulus ($E_{apparent}=298$) based on a solid cross section is lower than the corrected geometry flexural modulus ($E_{geo}=665$) and the mass ratio flexural modulus ($E_{m\phi }=672$) (Figure 9). Additionally, we see a lower coefficient of variation in the results calculated by the mass ratio (21%) compared to results calculated by geometry (23%). The apparent flexural modulus of a solid rectangular beam is determined by the second moment of the area and the distance from the neutral axis. In contrast, the flexural modulus of a rectangular hollow beam is influenced by the difference in the second moment of area between the solid and hollow sections. This difference accounts for the varying stress distributions across the cross-section due to the presence of the hollow section, correctly calculating higher stresses on the remaining material. The geometry and reduced mass of a hollow rectangular section affects the bending strength and load-carrying capacity of the beam, making it essential to consider both the solid and hollow sections when selecting an optimal print infill pattern. Summaries of the flexural strength and modulus as calculated by the three methods are shown in Table 6.

**Figure 10:**
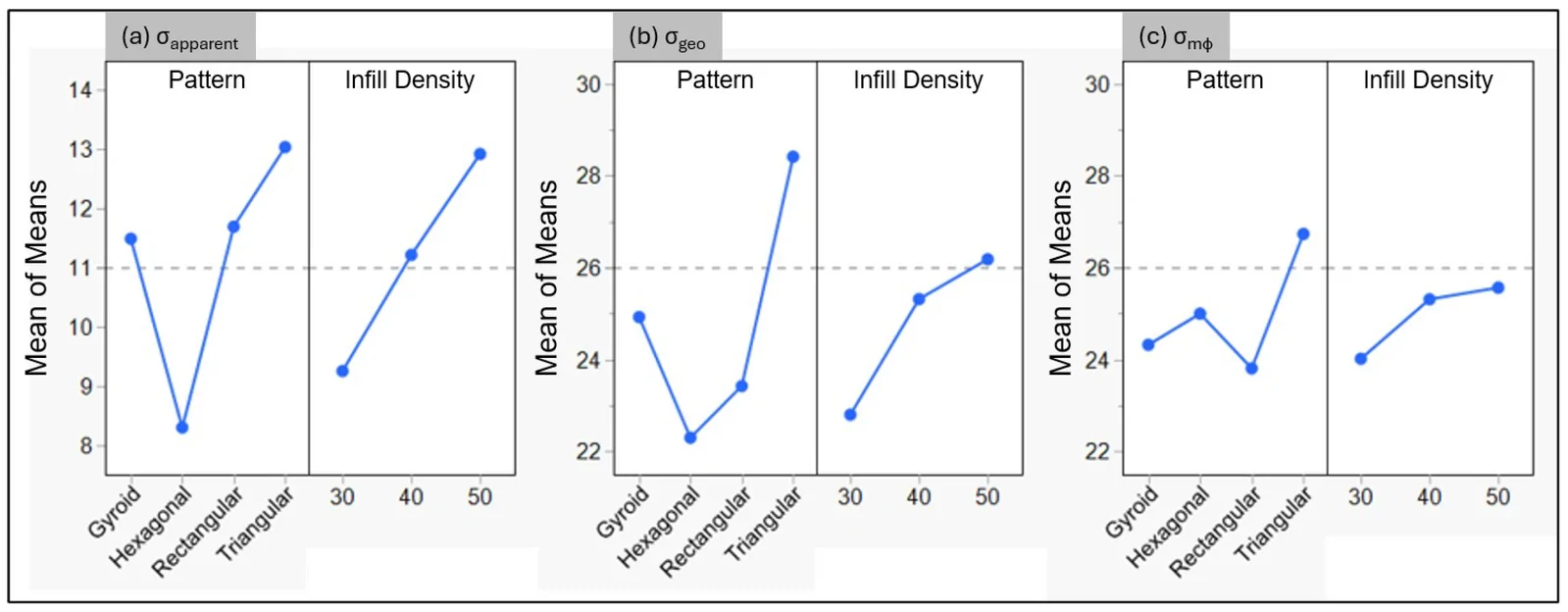
Main effect plots for means of (**a**) $\sigma_{apparent}$, (**b**) $\sigma_{geo}$, and (**c**) $\sigma_{m\phi }$.

Microstructural analysis using optical microscopy revealed features that govern the mechanical behavior of the printed samples. The short chopped carbon fibers remain highly oriented with the direction of material deposition (Figure 10).

The rectangular infill pattern exhibits a repeating orientation of the deposition of ±45° between successive layers, producing an orthogonal extrusion arrangement with frequent filament intersections. The chopped carbon fibers are aligned primarily along the extrusion direction, resulting in alternate 45° and −45° fiber orientations throughout the structure. The gyroid infill pattern generates a continuous, non-orthogonal deposition path such that the extrusion direction of successive layers varied continuously throughout the structure, producing a spatially changing fiber orientation. The triangular infill pattern consists of repeated 60° deposition orientations that form interconnected triangular cells. Multiple extrusion overlap regions and intersection points were observed as a result of the converging filament paths during printing within the triangular geometry. The hexagonal infill pattern exhibits a periodic honeycomb cellular structure with incline deposition paths connected at approximately 120° internal angles. Compared with the other patterns, the hexagonal geometry contains larger internal void regions and fewer extrusion intersection points.

**Figure 11:**
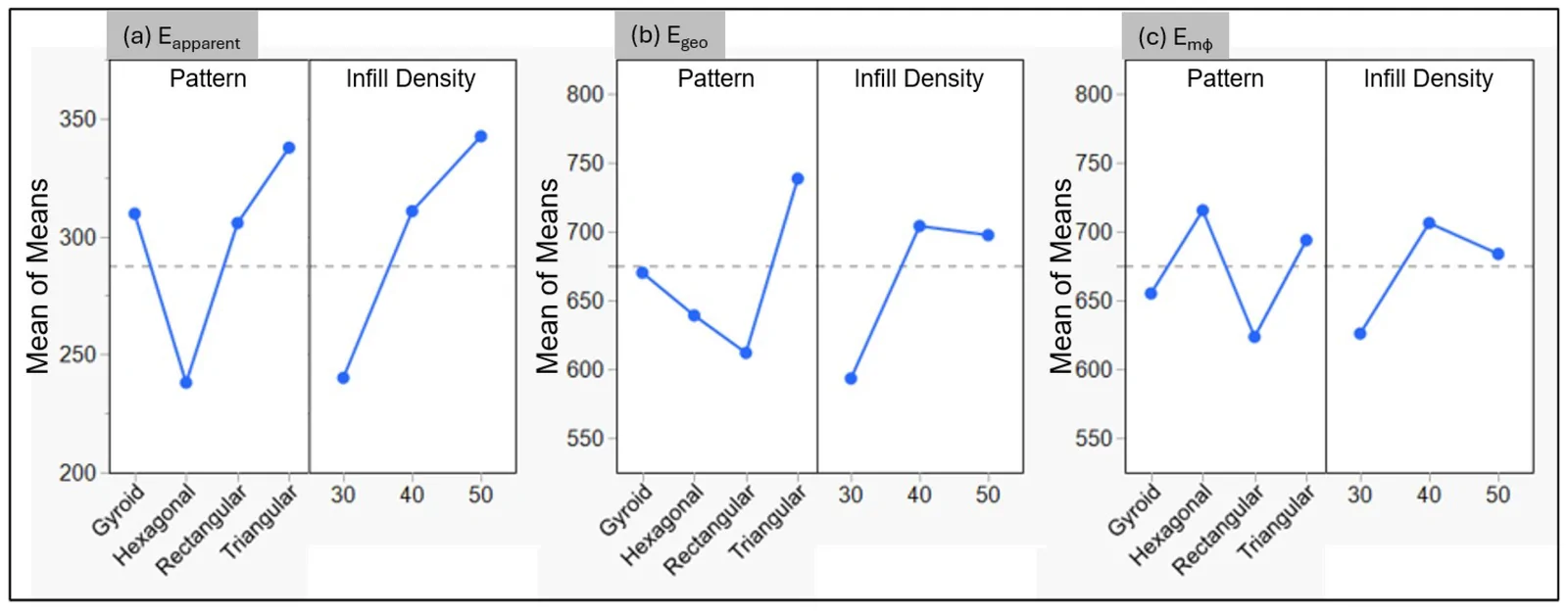
Main effect plots for means of (**a**) $E_{apparent}$, (**b**) $E_{geo}$, and (**c**) $E_{m\phi }$.

**Figure 12:**
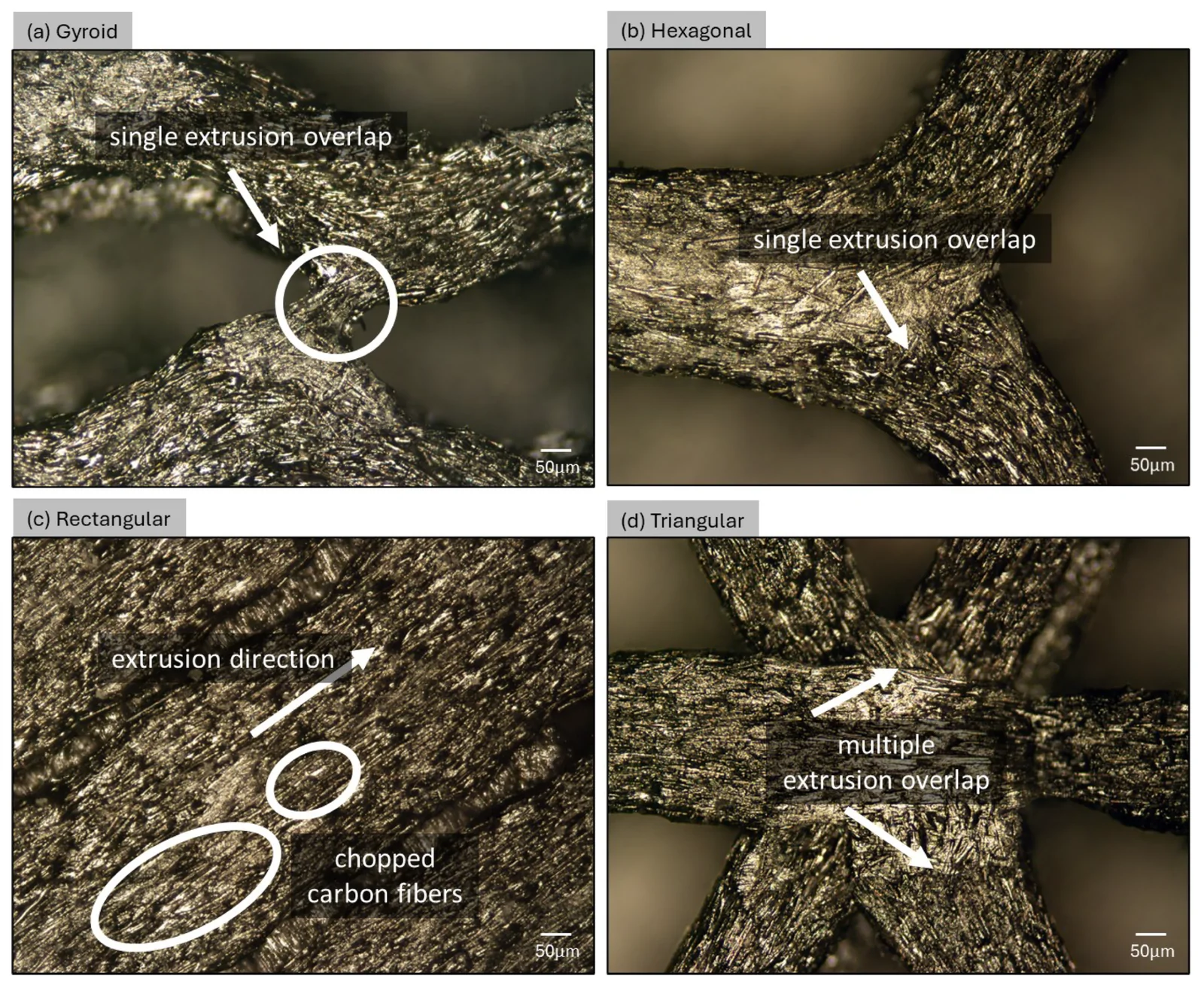
Microstructure of the Onyx samples under an optical microscope. (**a**) is printed in the gyroid pattern, (**b**) is printed in the hexagonal pattern, (**c**) is printed in the rectangular pattern, and (**d**) is printed in the triangular pattern.

Cross-sectional SEM images taken perpendicular to the printing direction revealed several microstructural defect features associated with the material extrusion process, including incomplete extrusion bonding, interlayer bonding regions, and internal void formations (Figure 11 and Figure 12). Evidence of a potential incomplete bonding between adjacent extrusions was observed in all infill patterns. The rectangular pattern showed frequent overlap between adjacent layers as a result of the alternating orthogonal deposition path, resulting in a higher density of observable interlayer contact regions. The gyroid and rectangular patterns displayed bonding between layers deposited in differing print orientations. In contrast, the hexagonal and triangular patterns primarily exhibited bonding between successive layers deposited in the same print direction. Additionally, the SEM images indicated that the chopped carbon fibers within the extruded filament were generally aligned along the deposition direction of each print path. Variations in fiber orientation between neighboring layers reflected the corresponding changes in extrusion orientation for each infill geometry.

**Figure 13:**
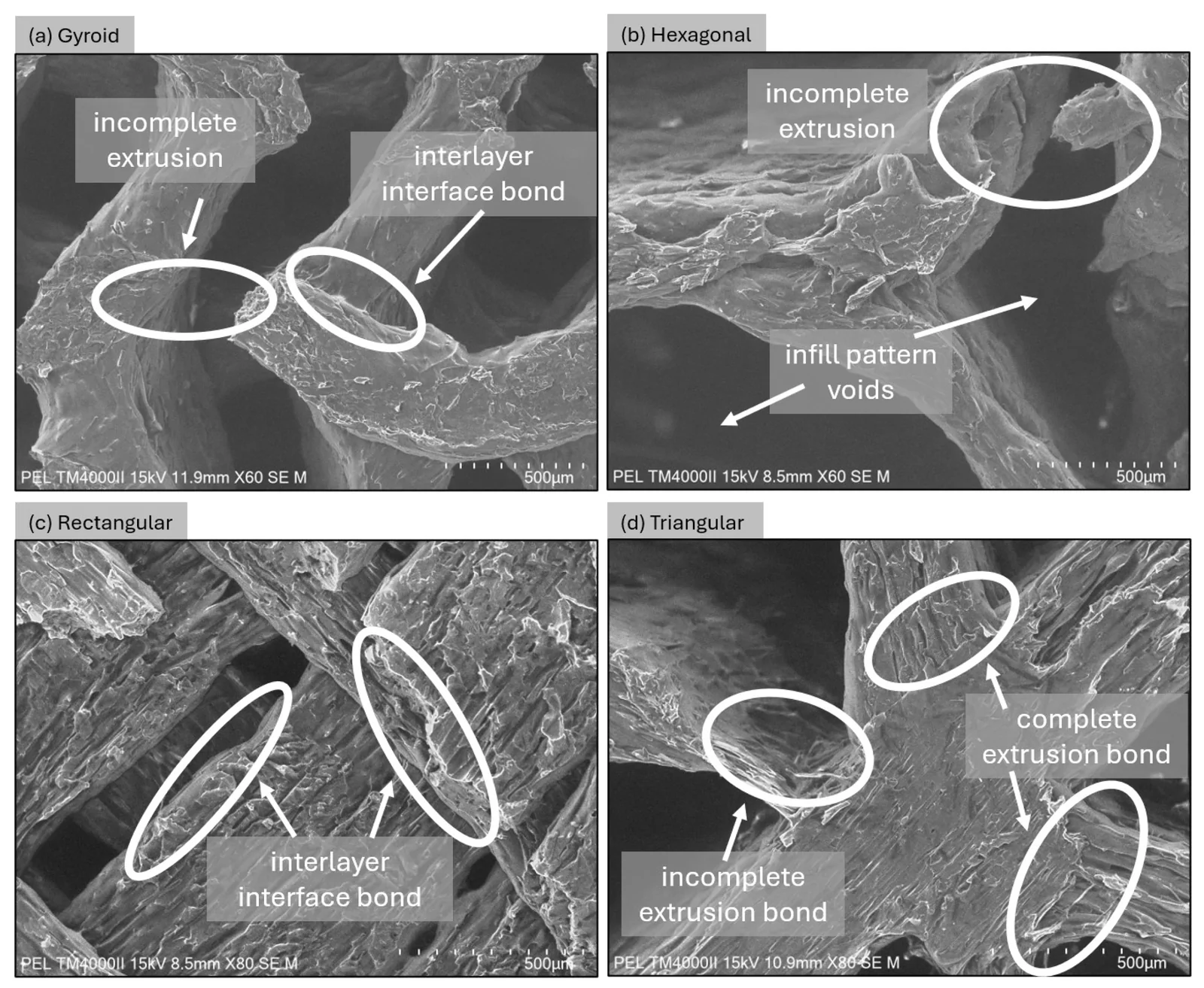
Microstructure of the Onyx samples under a SEM. (**a**) is printed in the gyroid pattern, (**b**) is printed in the hexagonal pattern, (**c**) is printed in the rectangular pattern, and (**d**) is printed in the triangular pattern.

**Figure 14:**
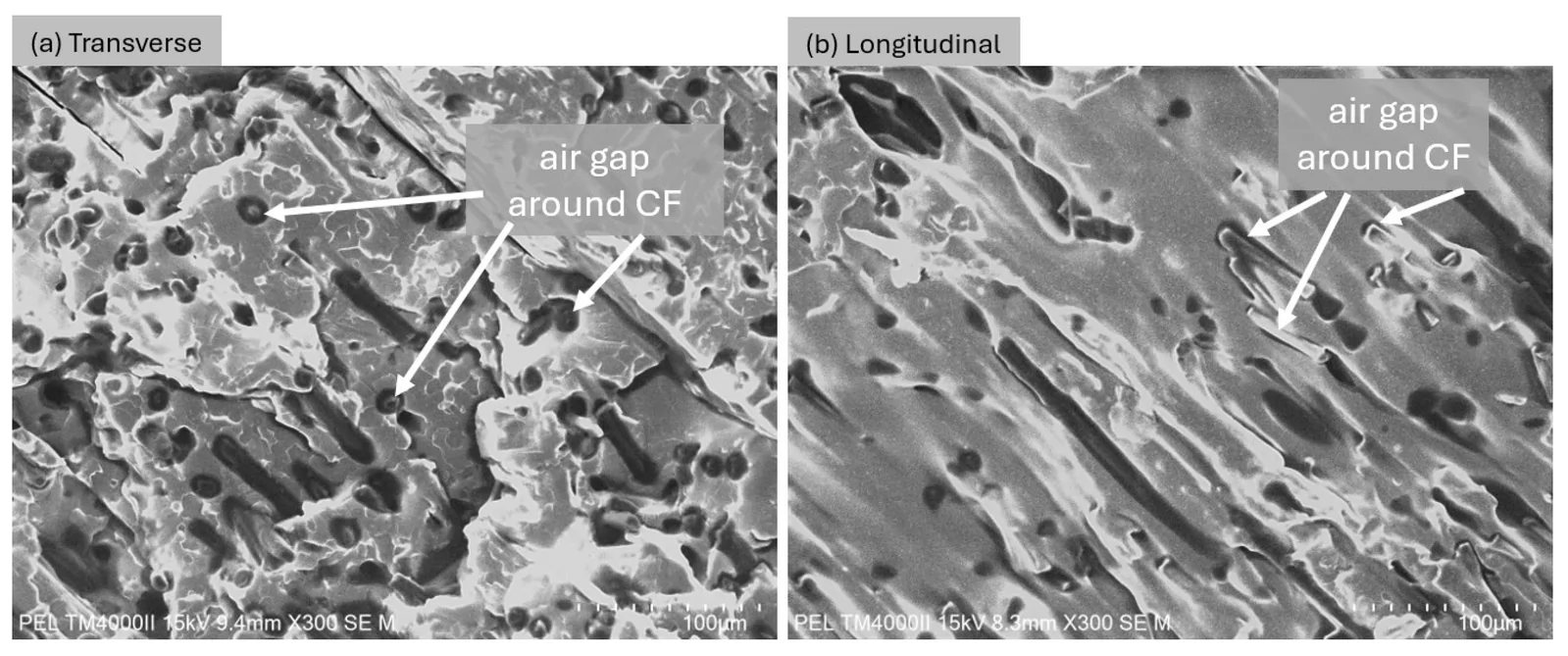
Microstructure of the Onyx samples under a SEM. (**a**) depicts a transverse cross-section of a sample fracture surface and (**b**) depicts a longitudinal cross-section of a sample.

An analysis of variance was carried out to evaluate the effects of infill pattern, infill density, and the interactions of both parameters on the measured flexural properties (Table 7). Main effect plots for the mean values are shown in Figure 13 and Figure 14. For the $\sigma_{apparent}$, both infill pattern and infill density were statistically significant factors (both *p*-values < 0.0001). Infill density produced the largest statistical effect (*F*-value = 21.49), followed by infill pattern (*F*-value = 19.31). The interaction between infill pattern and infill density was not significant (*p*-value = 0.0823).

After applying the geometry-based correction for flexural strength ($\sigma_{geo}$), both infill pattern and infill density remained statistically significant. However, the interaction between the two factors was not significant (*p*-value = 0.2003). When considering the mass-based correction for flexural strength ($\sigma_{m\phi }$), neither the infill pattern or infill density had a statistical significant effect. In addition, the interaction between the two variables was not significant for both the geometry-based (*p*-value = 0.2003) and mass-based (*p*-value = 0.6904) correction methods for the flexural strength.

The flexural modulus results exhibited similar trends. For the apparent flexural modulus ($E_{apparent}$), both infill pattern and infill density were statistically significant (both *p*-values < 0.0001), while the interaction of both factors was not significant (*p*-value = 0.4970). For the geometry-based correction for flexural modulus ($E_{geo}$), infill density remained significant, whereas the infill pattern (*p*-value = 0.1109) and interaction of both factors (*p*-value = 0.6643) were not statistically significant. The mass-based correction method for flexural modulus ($E_{m\phi }$), neither of the investigated factors, nor their interaction exhibited statistical significant (all *p*-values > 0.05).

The ANOVA results indicate that infill pattern and infill density significantly influence the apparent mechanical properties of the printed specimens. As correction methods accounting for effective load-bearing material were applied, the statistical significance of these factors decreased, and neither factor remained significant for the mass-based corrected flexural strength or modulus.

## 4. Discussion

In this study, we investigated the infill pattern and infill density printing parameters and their effects on mechanical properties. Flexural testing by three-point bending was performed to compare cross-sectional geometries and flexural properties. In addition, post-printing mass and dimensional measurements were evaluated for printing accuracy.

The post-print mass measurements demonstrate that the Eiger slicing software consistently overestimates the amount of material deposited during fabrication, regardless of pattern or infill density. The hexagonal pattern exhibited the largest post-print mass discrepancy of about 10% lower than the predicted mass, while the gyroid, rectangular, and triangular patterns had final print masses of about 8% lower than their Eiger predicted masses. The relatively consistent mass reduction observed for all patterns indicates that these discrepancies are systematic rather than random, suggesting an inherent limitation in the prediction model used by the slicer. This lower mass should be considered when forecasting the strength of a printed part.

All specimens were manufactured with identical external dimensions and showed minimal deviations from their intended geometry. This indicates that the Markforged printing system maintains high positional accuracy during printing. For all specimens, the lengths exhibited a slight increase, while the width and depth dimensions decreased. The variation in length in the specimens remained essentially unchanged, suggesting that the dimensional inaccuracies may be associated with extrusion geometry rather than warping or distortion of the entire part. The average deviations for the widths and depths collectively are approximately 0.5% and 3% reduced dimensions, respectively, from the intended geometry. The shrinkage of the width and depth dimensions could be attributed to the amount of extruded material deposited during the printing process due to the viscosity of the molten filament, the size/shape of the nozzle and the filament extrusion mechanism [36].

When subtracting outer layers and accounting for the interior infill of the specimens, the gyroid, rectangular, and triangular patterns exhibited a fairly accurate mass ratio for their selected infill densities. Our calculated percent infill was generally a little lower than the selected infill setting; however, there may be some inaccuracies in our calculations, as they are based on the reported density value of Onyx (1.2 g/cm^3^), and a slightly lower density would increase the calculated percent of infill. However, small differences in density would not be able to explain the drastic difference between our values and the requested values of infill percent prevalent for the hexagonal pattern, which exhibited the lowest effective infill percentage and the corresponding lower flexural strength and stiffness. One surprising result was that even when the desired infill percentage for the hexagonal pattern was increased from 40% to 50%, we actually saw a drop in effective infill percent from 22% to 21%. These results demonstrate that mechanical performance is governed by the actual deposited material rather than the selected infill percentage alone, emphasizing the importance of experimentally validating the effective infill.

The analysis of the flexural strength values obtained for a material with a solid fill percentage of 100% gave an average of 36.44 MPa. This is significantly less than the value according to the material data sheet for Onyx reported by Markforged in Table 1 (71 MPa) [18]. It is unclear as to the source of this discrepancy. Markforged states that the measured method is similar to ASTM D790, but does not report details of the manufacturing process or parameters of their test specimens. It can also be noted that even though parameters were set for a solid part in our work, the measured mass of the part indicated that the total infill percent of the part was closer to 83%, which might explain the differences in the reported strength. However, our values are similar to those in similar unreinforced Onyx flexural studies by Maier et al. [29] who reported a flexural strength value for a part printed with a solid fill percentage of 100% of 40.16 MPa, and greater than those reported by Ramachandran et al. [31] (10 MPa). However, the study by Ramachandran et al. does not report the build direction of their tested parts, which could be a major factor in calculating the strength of the material of these tests.

After correcting for reduced material in the 30%, 40% and 50% infill patterns, part strength calculated using both geometric and mass ratio based methods produced remarkably similar strengths with a mean of just under 25 MPa. It is interesting that despite this consistency, the material strength calculated from the partially filled specimens was lower than the solid reference. This suggests that the loss in mechanical performance cannot be attributed solely to the reduction in material. Micrographs of the parts indicate discontinuous filaments that result in incompletely interrupted extrusions and extrusion bonds that reduce the efficiency of stress transfer throughout the structure. This could result in stress concentrations that lead to early failure compared to those predicted by beam theory. Although these incomplete extrusions can occur during any time, no matter the infill percentage, they may have a larger effect for partial infills than for a solid printed part. Although our correction methods account for differences in the cross-sectional properties, reducing variability and getting closer to the true material strength, they cannot fully compensate for the structural effects introduced by the infill geometry. The solid specimens, therefore, represent the upper limit of flexural performance achievable under experimentally selected printing conditions. A more detailed study of the rectangular infill from 0% up to 92% may reveal where and how the strength may transition from this lower value of partial infills to the larger value of a solid printed part.

Comparison of the flexural properties calculated using the geometry-based correction method with those obtained using the mass-based correction demonstrates that the actual amount of deposited material is a more consistent predictor of a singular material flexural strength and modulus than the selected infill density alone. Although both correction methods produced similar mean values for flexural strength and modulus, the mass-based approach consistently exhibited a lower coefficient of variation, indicating that it more effectively accounted for the differences from specimen-to-specimen introduced during the printing process. Since both the strength and stiffness of the part are directly influenced by the effective load-bearing material, normalizing the measured properties using the post-print mass ratio reduced the variability associated with inconsistencies in material deposition, extrusion efficiency, and internal void content. In contrast, geometry-based corrections rely on idealized cross-sectional representations that may not fully capture any manufacturing printing-induced deviations from the intended infill architecture. The variances in the geometry-based method could also be attributed to the miscalculation of the extrusion widths of different segments that are dependent on the amount of filament extruded and the speed at which extrusions solidify.

Classic beam theory would suggest that the hexagonal and triangular patterns should exhibit stronger flexural properties because they are characterized by vertically repeating cellular geometries in which the filament is maintained from one layer to the next. This repeated stacking produces a continuous load-bearing structure through the thickness of the specimen, allowing the material to contribute more consistently to bending resistance. The triangular geometry forms a repeating truss-like structure with numerous filament intersections that provide multiple pathways for stress redistribution while the hexagonal pattern forms a honeycomb-like structure with reduced filament intersections. In contrast, the gyroid and rectangular patterns redistribute the filament paths between successive layers through orthogonal or continuously curved deposition extrusions. As a result, the internal structure does not maintain the same continuous through-thickness alignment, reducing the efficiency with which material located away from the neutral axis contributes to bending resistance. However, following the mass-based correction, the infill patterns are indistinguishable from one another and the differences in flexural strengths disappear. Therefore, the only reason one pattern may appear stronger than another pattern, is because more material was deposited during fabrication. This is why the triangular pattern exhibited the strongest flexural properties while the hexagonal pattern exhibited the weakest. This suggests that the uniform distribution of material throughout the specimen assumption provides a better prediction of part behavior than the geometry-based method.

These findings suggest that the mass-based correction provides a practical method for determining the effective structural material strength and can be used to predict part strength and stiffness with more accuracy than more complex geometry-based models. Consequently, a simple mass-based correction offers an efficient and effective alternative for estimating the mechanical properties of FFF manufactured parts while avoiding the time and computational efforts associated with detailed geometric characterization or finite element analysis.

Carbon fibers help alleviate bending stressors within the nylon matrix; however, SEM micrographs show that there are air gaps around the short chopped carbon fibers and the nylon matrix. The air pores throughout the carbon fibers suggest that they could be the result of poor adhesion of nylon to the carbon fibers after extrusion solidification. This may suggest that there may be poor adhesion of the short chopped carbon fibers to the matrix during the filament melting–extrusion–solidification process that occurs during FFF printing. Future studies may include how the cooling rate affects the short chopped carbon fibers from bonding with the nylon matrix or if any methods of post-process heat treatment can enhance the mechanical properties of a printed part.

Several limitations of this study should be acknowledged when interpreting the results. First, all specimens were fabricated using a single material, a nylon-based Onyx by Markforged. Nylon is characterized by its flexible properties which reduces stress concentrations making it better for uniform distribution while brittle materials may not behave this way. In addition, nylon is hygroscopic and the amount of moisture in the material can affect its mechanical properties. Secondly, all specimens were fabricated using a single printer, the Markforged Mark Two. Material extrusion 3D printing is notoriously finicky, so factors such as wear on the printer/nozzle, leveling methods, bed temperatures, etc., might produce different quality specimens with different strengths. Lastly, the printing parameters utilized in this study (Table 2) may produce different flexural properties to other studies with differing printing parameters/conditions.

## 5. Conclusions

The findings of this study help support the overall development of incorporating bio-based fibers into engineered composites to improve a base material’s properties [37]. These engineered composites are crucial for advancing engineering applications such as tooling, manufacturing fixtures, robotics, functional prototypes, and low-volume end-use parts because these applications require components that provide sufficient mechanical performance while minimizing weight, material usage, and printing time.

This work investigated the influence of infill pattern and infill density setting using Markforged Eiger slicing software and their effects on the flexural behavior and microstructural characteristics of Markforged Onyx composites fabricated through FFF using the Mark Two printer.

- Our work determined a material strength of 36.44 MPa and stiffness of 780.95 MPa for a part printed in the solid infill pattern with 100% infill density, consistent with the work by Maier et al. [29] and Martulli et al. [28].
- The Eiger software consistently over estimated the amount of material that would be deposited for each part.
- For gyroid, rectangular, and triangular infill patterns, the Eiger software typically over predicted the measured infill percentage, but was always within a few percent. For the hexagonal infill pattern, the Eiger software significantly over predicted the infill percent by a factor of 2 or more, indicating a significant calculation error.
- Parts were typically oversized in length by less than 0.1 percent, undersized in width by less than 1 percent, and undersized in depth by about 2–4 percent.
- For a given selected infill density, the rectangular and triangular infill geometries provided the strongest flexural strength and stiffest modulus, while the hexagonal pattern exhibited reduced performance due to larger internal voids, lower effective mass, and poor printing predictions from the Eiger software.
- After correcting for amount of material deposited, we found no significant difference between infill patterns and infill densities, demonstrating that part strength is mainly dictated by the amount of material deposited.

Future work may focus on improving fiber–matrix adhesion, reducing void formation during extrusion, the effects of microstructural properties such as crystallinity on material properties, and investigating post-processing or processing parameter modifications to further enhance the mechanical reliability of carbon fiber–reinforced polymer components produced by FFF.

## Figures and Tables

**Table 1:** Mechanical properties of the Onyx composite [18].

Material Property		
Tensile Modulus	2.4	(GPa)
Ultimate Tensile Stress	40	(MPa)
Flexural Strength	71	(MPa)
Flexural Modulus	3.0	(GPa)
Izod Impact - notched	330	(J/m)
Heat Deflection Temperature	145	(°C)
Density	1.2	(g/cm^3^)

**Table 2:** Fixed and varying experimental printing parameters.

Fixed Experimental Parameters		
Nozzle Diameter	0.4	(mm)
Bed Orientation	Flat	-
Infill Angle	45	(°C)
Layer Height	0.125	(mm)
Roof and Floor Layers	1	-
Wall Layers	1	-
Variable Experimental Parameters		
Infill Density	30, 40, 50	%
Infill Pattern	Gyroid, Hexagonal	-
	Rectangular, Triangular	

**Table 3:** Sample prep polishing procedure indicating speed in rotations per minute (RPM) and directions clockwise (CW) or counterclockwise (CWW).

Step	Abrasive/Surface	Table Speed (RPM)	Spindle Speed (RPM)	Time
1	180 grit SiC paper	100 CCW	100 CCW	Until Plane
2	400 grit SiC paper	100 CW	100 CW	Until Plane
3	800 grit SiC paper	150 CCW	150 CCW	1 min
4	1200 grit SiC paper	150 CW	150 CW	1 min
5	3 μm polycrystalline diamond	200 CCW	200 CCW	3 min

**Table 4:** Mass measurement averages for all samples.

	Part				Infill			
Infill Pattern/	$m_{eiger}$	$m_{part}$	error	mϕ	$m_{eiger}$	$m_{infill}$	error	mϕ
Infill Density	(g)	(g)	(%)	(%)	(g)	(g)	(%)	%
Shell/Solid								
0%	1.11	1.04	−6.33	0.17	-	-	-	-
100%	5.93	5.17	−12.82	0.83	4.82	4.13	−14.32	0.81
Gyroid								
30%	2.67	2.46	−7.80	0.40	1.56	1.42	−8.84	0.28
40%	3.18	2.90	−8.73	0.47	2.07	1.86	−10.02	0.36
50%	3.67	3.40	−7.38	0.55	2.56	2.36	−7.83	0.46
Hexagonal								
30%	2.06	1.88	−8.92	0.30	0.95	0.84	−11.94	0.16
40%	2.44	2.15	−11.82	0.35	1.33	1.11	−16.39	0.22
50%	2.24	2.11	−5.71	0.34	1.13	1.07	−5.09	0.21
Rectangular								
30%	2.86	2.58	−9.80	0.42	1.75	1.54	−12.0	0.30
40%	3.34	3.01	−9.94	0.49	2.23	1.97	−11.74	0.38
50%	3.85	3.49	−9.48	0.56	2.74	2.45	−10.75	0.48
Triangular								
30%	2.87	2.61	−8.92	0.42	1.76	1.57	−10.55	0.31
40%	3.19	2.96	−7.21	0.48	2.08	1.92	−7.67	0.37
50%	3.63	3.40	−6.42	0.55	2.52	2.36	−6.46	0.47

**Table 5:** Dimensional measurement averages for all samples.

Infill Pattern/	*l*	Error	*B*	Error	*D*	Error	$\bar{b}$	*d*	$I_{apparent}$	$I_{geo}$	$I_{m\phi }$
**Infill Density**	**(mm)**	**(%)**	**(mm)**	**(%)**	**(mm)**	**(%)**	**(mm)**	**(mm)**	**(mm^4^)**	**(mm^4^)**	**(mm^4^)**
Shell/Solid											
0%	127.08	0.06	12.42	−2.17	3.19	−0.44	-	-	33.48	-	5.62
100%	127.15	0.11	12.60	−0.77	3.07	−4.00	-	-	30.46	-	26.30
Gyroid											
30%	127.15	0.12	12.61	−0.71	3.11	−2.94	9.52	2.86	31.49	13.00	12.52
40%	127.12	0.10	12.61	−0.72	3.08	−3.63	8.72	2.83	30.83	14.28	14.44
50%	127.14	0.11	12.59	−0.90	3.13	−2.25	8.05	2.88	32.10	16.10	17.62
Hexagonal											
30%	127.12	0.09	12.58	−0.98	3.10	−3.13	10.63	2.85	31.23	10.72	9.46
40%	127.13	0.10	12.60	−0.82	3.10	−3.25	10.15	2.85	31.16	11.66	10.83
50%	127.12	0.10	12.59	−0.83	3.10	−3.06	9.75	2.85	31.35	12.49	10.70
Rectangular											
30%	127.14	0.11	12.57	−1.01	3.13	−2.31	8.97	2.88	32.02	14.22	13.35
40%	127.12	0.09	12.62	−0.60	3.07	−4.13	8.22	2.82	30.39	15.05	14.77
50%	127.13	0.10	12.65	−0.39	3.08	−3.62	7.40	2.83	30.93	16.89	17.42
Triangular											
30%	127.14	0.11	12.59	−0.85	3.15	−1.69	9.39	2.90	32.67	13.66	13.79
40%	127.13	0.11	12.61	−0.74	3.12	−2.38	9.01	2.87	32.10	14.22	15.32
50%	127.13	0.10	12.59	−0.88	3.13	−2.19	8.04	2.88	32.17	16.17	17.66

**Table 6:** Numerical flexural strength and modulus results of apparent and corrected three-point bending test averages for all samples.

Infill Pattern/	$\sigma_{apparent}$	$\sigma_{geo}$	$\sigma_{m\phi }$	$E_{apparent}$	$E_{geo}$	$E_{m\phi }$
**Infill Density**	**(MPa)**	**(MPa)**	**(MPa)**	**(MPa)**	**(MPa)**	**(MPa)**
Shell/Solid						
100%	36.44	-	-	780.95	-	-
Gyroid						
30%	9.34	22.57	23.44	248.32	601.40	624.37
40%	11.08	23.99	23.71	318.78	687.95	680.03
50%	14.04	28.22	25.82	361.84	720.95	658.43
Hexagonal						
30%	7.37	21.42	24.28	196.19	571.32	648.00
40%	9.35	24.97	26.90	282.93	755.89	750.98
50%	8.17	20.51	23.82	235.04	590.02	689.07
Rectangular						
30%	9.28	20.90	22.30	235.53	529.89	562.55
40%	11.52	23.26	23.74	309.47	624.67	632.69
50%	14.28	26.11	25.40	372.07	681.14	659.05
Triangular						
30%	11.04	26.32	26.06	280.39	670.47	664.51
40%	12.88	29.04	26.91	331.71	747.40	693.20
50%	15.2	29.89	27.23	400.75	797.41	734.13
Mean	11.13	24.77	24.97	297.75	664.88	671.91
Standard Deviation	2.90	4.50	3.41	85.34	152.01	139.89
Coefficient of Variation	0.26	0.18	0.14	0.29	0.23	0.21

**Table 7:** ANOVA analysis of flexural strength and modulus results.

Source	DF	SS	MS	*F*-Value	*p*-Value	Remarks
$\sigma_{apparent}$						
Infill Pattern	3	181.97	60.66	19.31	<0.0001	Significant
Infill Density	2	134.98	67.49	21.49	<0.0001	Significant
Both	6	37.92	6.32	2.01	0.0823	Insignificant
$\sigma_{geo}$						
Infill Pattern	3	317.99	105.99	7.81	0.0002	Significant
Infill Density	2	123.34	61.67	4.54	0.0156	Significant
Both	6	121.69	20.28	1.49	0.2003	Insignificant
$\sigma_{m\phi }$						
Infill Pattern	3	73.16	24.39	2.12	0.1102	Insignificant
Infill Density	2	27.51	13.75	1.19	0.3116	Insignificant
Both	6	44.84	7.47	0.6493	0.6904	Insignificant
$E_{apparent}$						
Infill Pattern	3	80,362.87	26,787.62	5.80	0.0018	Significant
Infill Density	2	109,735.84	54,867.92	11.88	<0.0001	Significant
Both	6	25,177.07	4196.18	0.9083	0.4970	Insignificant
$E_{geo}$						
Infill Pattern	3	133,636.67	44,545.56	2.11	0.1109	Insignificant
Infill Density	2	154,255.22	77,127.61	3.66	0.0332	Significant
Both	6	86,375.16	14,395.86	0.68	0.6643	Insignificant
$E_{m\phi }$						
Infill Pattern	3	74,870.923	24,956.97	1.22	0.3117	Insignificant
Infill Density	2	68,612.098	34,306.05	1.6808	0.1970	Insignificant
Both	6	51,006.921	8501.15	0.4165	0.8644	Insignificant

## Data Availability

The raw data supporting the conclusion of this article will be made available by the authors, without undue reservation.

## Abbreviations

The following abbreviations are used in this manuscript:

ANOVA	Analysis of variance
ASTM	American Society for Testing and Materials
CW	Clockwise
CWW	Counterclockwise
DF	Degree of freedom
FFF	Fused filament fabrication
ISO	International Organization for Standardization
MS	Mean of squares
OM	Optical microscope
PLA	Polylactic acid
RPM	Rotations per minute
SEM	Scanning electron microscope
SS	Sum of squares
